# Study on Online Correction of Polished Rod Dynamometer Cards and Digitalization Application of Pump Dynamometer Cards

**DOI:** 10.3390/s25082392

**Published:** 2025-04-09

**Authors:** Hukun Yang, Jianhua Ma, Yongqin Dai, Junmin Jia, Yu Lu, Xiyu Zhang, Ruihui Hao

**Affiliations:** 1 School of Mechanical Science and Engineering, Northeast Petroleum University, Daqing 163318, China; hk_yang@163.com (H.Y.);; 2 Oil Production Technology Research Institute of Liaohe Oilfield, Petrochina, Panjin 124010, China

**Keywords:** polished rod dynamometer card correction, pump dynamometer card, one-dimensional wave equation difference solution, production and liquid level calculation

## Abstract

The polished rod dynamometer operates under alternating loads and large temperature differences for a long time, inevitably leading to zero drift and temperature drift issues. At the same time, conventional inversion of polished rod dynamometer cards fails to consider the impact of friction loads, resulting in inaccurate production and liquid level calculations from pump dynamometer cards. Based on the oil-filled environment in the sucker rod and tubing during the upstroke of the pumping unit, this paper proposes a rapid identification method for the four characteristic points of the polished rod dynamometer card to obtain a calculation method for friction loads at the velocity reversal points A and C. The gravity of the polished rod string in the liquid column serves as the benchmark for calibrating the polished rod dynamometer card. Combined with basic well data, a one-dimensional wave equation difference calculation method is used to solve for the pump dynamometer card. An approximation algorithm is employed to achieve rapid calibration of the polished rod dynamometer card and inversion of the pump dynamometer card. Calculation and engineering application results indicate that the accuracy of production and liquid level calculations obtained from the pump dynamometer card through online correction of the polished rod dynamometer card exceeds 90%, achieving the goal of engineering digitization applications.

## 1. Introduction

The dynamometer card (DC) for a beam pump well represents the load distribution at the polished rod suspension point during a complete stroke [1]. The horizontal axis corresponds to the suspension point displacement, while the vertical axis represents the suspension point load. The DC serves as a direct representation of the surface system and downhole conditions of a beam pump well and is a critical “intermediate node” for analyzing and studying the system [2]. Currently, oil wells primarily use load sensors and displacement sensors to measure the polished rod DC [3,4,5]. However, due to the load sensors being subjected to alternating loads over extended periods and operating in harsh field environments [6], their reliability tends to degrade. This degradation can lead to zero drift and temperature drift, resulting in errors in the DC acquisition. These errors increase maintenance workload and costs. Low accuracy of DCs not only affects the correct analysis and judgment of some well conditions but also makes it difficult to meet the demands of modern oil well digitization.

Converting the polished rod DC into the downhole pump dynamometer card has consistently been a hot research topic for oilfield scientists during the production process of beam pump wells [7]. The downhole pump DC forms the foundation for accurately calculating oil well production and determining the dynamic fluid level, and it is also a critical basis for the digitization of beam pump wells. Currently, most domestic and international researchers adopt the difference method or Fourier series method to solve the Gibbs [8] one-dimensional wave equation in their studies on converting the polished rod DC to the pump DC [9,10,11]. However, during their research, scholars often assume that the polished rod DC measurements are accurate (i.e., ignoring issues such as zero drift and temperature drift in dynamometers) and neglect the friction load in the downhole system [12,13,14].

The presence of zero drift, temperature drift, and the proportion of friction load in the polished rod load directly determines the accuracy of converting the polished rod DC to the downhole pump DC. For instance, in cases of downhole paraffin deposition or overly tight wellhead stuffing box seals, the downhole pump DC obtained using traditional conversion methods will lack validity. Friction between the rod and fluid is an unavoidable phenomenon during the operation of beam pump wells. Therefore, using traditional methods to solve the one-dimensional wave equation for obtaining the downhole pump DC often leads to inaccuracies in well production and dynamic fluid level calculations.

This study establishes a mathematical model for converting the polished rod DC to the downhole pump DC, based on the assumption that the load sensor rod in the polished rod dynamometer remains elastic. By combining the structural parameters of the beam pump and downhole operating conditions, the model is solved using the difference method to obtain the downhole pump DC.

A rapid identification method for the four characteristic points (A, B, C, D) of the polished rod DC is proposed, using the rate of change in the polished rod load. Points A and C on the polished rod DC are identified as the velocity reversal points of the polished rod. Based on this, the friction load is recognized and eliminated from the original DC, mitigating the impact of the friction load on the polished rod DC.

Additionally, using the principle of load measurement with a load sensor, the load of the rod string within the well fluid during the downstroke of the beam pump serves as a reference parameter for correcting the polished rod DC. An iterative algorithm is employed to set a predicted value for the dynamic fluid level in the well. This allows for the iterative calculation of the downhole pump DC to obtain the computed dynamic fluid level. When the difference between the predicted and computed dynamic fluid levels falls within a specified error range, the load sensor’s zero-adjustment coefficient and the elasticity coefficient of the sensor rod can be calibrated accordingly.

In oilfield engineering, dynamic fluid level measurement sensors are installed in the oil casing annulus, while wellhead metering tanks are utilized to accurately measure both the dynamic fluid level and production rates of oil wells. This study conducted a comparative analysis and achieved a measurement accuracy exceeding 90%. This approach addresses the issues typically associated with traditional methods, such as friction loads, zero drift, and temperature drift affecting load sensors used to convert polished rod DCs into downhole pump DCs for production and fluid level measurements [15,16,17,18]. These findings offer valuable theoretical and practical foundations for engineering applications and future deployments.

## 2. Analysis of Measurement Errors in Beam Pump Polished Rod Dynamometer Cards

The polished rod DC of a beam pump well consists of the polished rod displacement and load. The displacement is obtained using a wire displacement sensor or an acceleration sensor through double integration, while the load is measured using a load sensor.

### 2.1. Load Error Analysis

**Figure 1:**
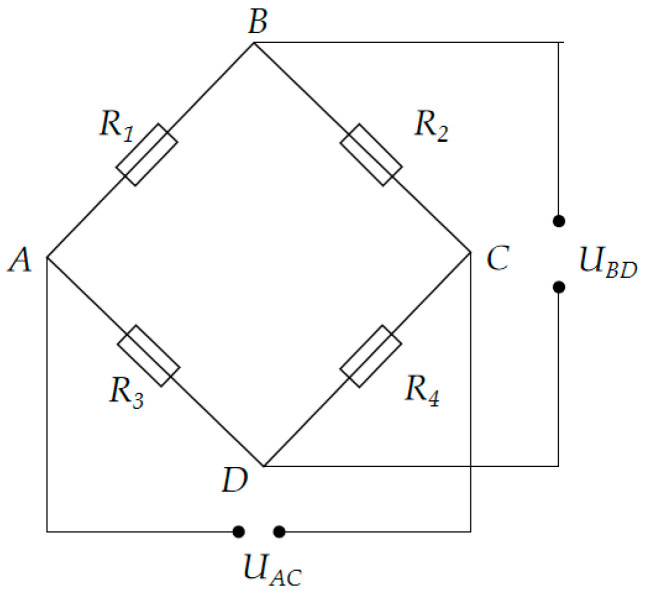
Resistance strain gauge load sensor.

Load sensors are widely used in various force measurement devices [19], particularly in beam pump dynamometers, where their core component is the strain gauge. The strain gauge is an inductive element made of resistance wire and is attached to an elastic element. When an external load is applied, the elastic element deforms, causing the strain gauge to deform as well, which leads to a change in its resistance value. To convert this change into an electrical signal output, a Wheatstone bridge circuit is typically used [20], as shown in Figure 1.

The input terminals A and C of the bridge are connected to the excitation voltage $U_{AC}$, while the output voltage $U_{BD}$ is measured across the output terminals B and D. The bridge output voltage $U_{BD}$ is:

(1) $$U_{BD}=\left(\frac{R_{1}}{R_{1}+R_{2}}-\frac{R_{3}}{R_{3}+R_{4}}\right)\cdot U_{AC}$$

In the ideal initial state, where no load is applied, the resistances $R_{1}=R_{2}=R_{3}=R_{4}$ are typically designed such that the bridge is in a balanced state, and the output voltage $U_{BD}=0$. When a load is applied, the resistance of the strain gauges changes, causing the bridge to become unbalanced, and the output voltage is no longer zero. The change in output voltage, determined by the degree of bridge imbalance, can accurately reflect the magnitude and direction of the load.

In the ideal initial state, where no load is applied, the bridge is typically designed to be in a balanced state, resulting in zero output voltage. When a load is applied, the resistance of the strain gauges changes, causing the bridge to become unbalanced, and the output voltage is no longer zero. The change in output voltage, based on the degree of imbalance in the bridge, can accurately reflect the magnitude and direction of the load.

Record the output voltage signal of the load sensor. When using a strain gauge sensor, the output signal is typically the voltage difference $U_{BD}$ from the Wheatstone bridge circuit. At the same time, record the corresponding known load values to establish the relationship between the load and the output signal.

(2) $$F=k\cdot U_{BD}+b$$

where F represents the actual load value; k represents the sensitivity coefficient; and b represents the zero-offset coefficient.

However, due to the influence of load sensor materials and design characteristics, load sensors may experience creep. Under alternating loads, repeated stress can lead to material fatigue and structural relaxation, especially in strain gauges. Additionally, changes in environmental temperature can significantly affect the resistance value of the strain gauge [21]. Under prolonged alternating load conditions, the temperature of the load sensor may fluctuate due to the internal damping effect of the materials, exacerbating sensor creep.

As a result, over the long-term use of load sensors, the coefficients k and b will change, causing a shift between the measured values and the actual values. In some cases, the measured values may no longer accurately reflect the actual load. Therefore, the periodic manual calibration method used for oil well dynamometers during operation presents significant challenges to production management. Research into online real-time correction of dynamometer load measurements is of great importance. The methods for correcting load measurement errors will be discussed in the analysis that follows.

### 2.2. Polished Rod Displacement Error Analysis and Correction

#### 2.2.1. Polished Rod Displacement Error Analysis

Polished rod DCs typically use wire displacement sensors or accelerometer sensors to measure displacement. When using a wire displacement sensor, displacement is measured through a metal cable. Prolonged reciprocating motion can lead to wear on the cable, and factors such as wind load and whether the cable is parallel to the polished rod can affect the accuracy of displacement measurement [22]. Accelerometer sensors measure the acceleration data of the polished rod [23,24,25], and then the displacement is obtained by double integration over time. However, cumulative integration errors (especially over long periods) can lead to drift in the displacement calculation. Even small acceleration signals can accumulate significant errors after multiple integrations, causing the measured displacement to deviate from the actual displacement [26,27,28].

**Figure 2:**
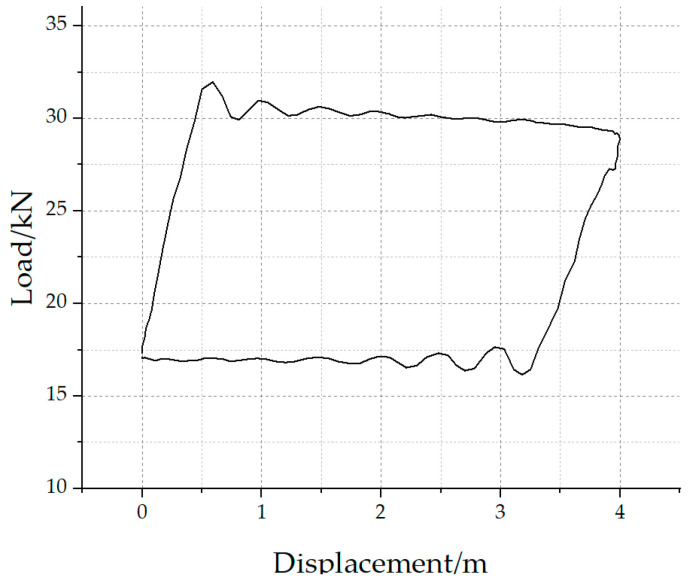
Measured polished rod dynamometer card.

As shown in Figure 2, the corresponding beam pump model is CYJY10-4.2-53HB (This pumping unit originates from Daqing Equipment Manufacturing Group in China). According to the specifications of this model, the maximum stroke of the polished rod is 4.2 m. However, the actual DC corresponds to a polished rod stroke of 4.0 m, which is caused by displacement measurement errors. A comparison between the measured displacement values in the actual DC and the structural parameters of the beam pump reveals that the displacement measurements in the DC often show discrepancies, with values either too large or too small compared to the actual measurements. Therefore, to accurately analyze the beam pump DC or invert the polished rod DC to a downhole pump DC, it is necessary to correct the polished rod displacement.

#### 2.2.2. Polished Rod Displacement Correction

According to the working principle of the beam pump, the primary motion mechanism is the crank-rocker mechanism, which converts the circular motion of the crank into the reciprocating linear motion of the horsehead between the top dead center (TDC) and bottom dead center (BDC). The kinematic characteristics of the four-bar linkage mechanism form the basis for determining the fundamental motion parameters of the beam pump. By establishing an accurate kinematic model of the polished rod based on the structural parameters of the beam pump, it is possible to correct the displacement of the polished rod.

**Figure 3:**
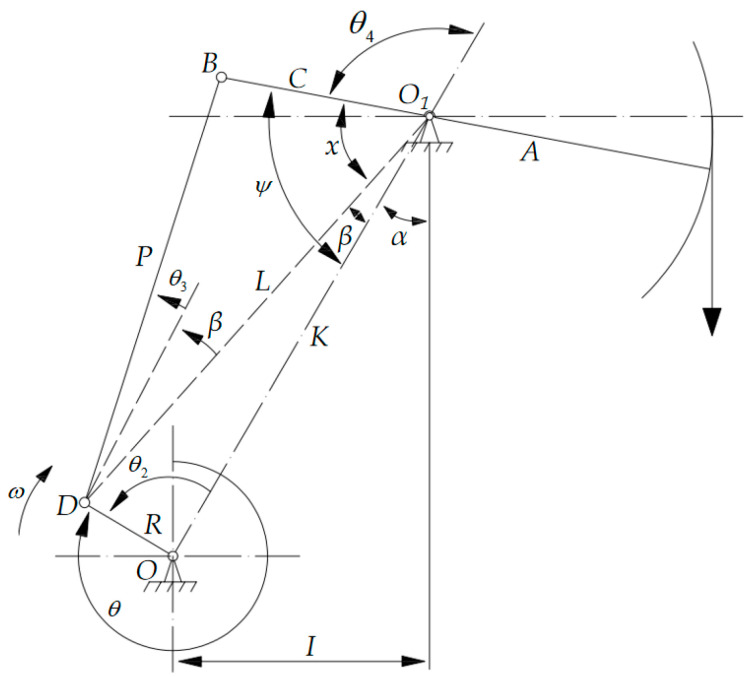
Schematic diagram of the beam pump mechanism.

As shown in the schematic diagram of the walking beam-type beam pump structure in Figure 3, the following angular directions are defined for analysis:

(1) The crank angle θ is measured starting from the 12 o’clock position, with positive values taken in the clockwise direction;
(2) The reference angle $\theta_{2},\theta_{3},\theta_{4}$ points for each link are measured starting from the base link $OO_{1}$, with positive values taken in the counterclockwise direction.

In Figure 3, R is the crank radius; P is the connecting rod length; C is the rear arm length of the walking beam; K is the base link length; A is the front arm length of the walking beam; I is the horizontal projection of the base link.

The BDCs are considered the zero displacement points, with the upward direction defined as the positive direction of displacement. The displacement at any moment is then expressed as:

(3) $$\left\{\begin{array}{l} \psi =x+\beta \\ \psi_{\max}=\cos^{-1}[\frac{C^{2}+K^{2}-(R+P{)}^{2}}{2CK}]\\ S=(\psi_{\max}-\psi )A \end{array}\right.$$

The method for calculating the motion laws of the polished rod, as described above, has been widely adopted. However, in dynamic analyses of the polished rod, it is often overlooked that the kinematic laws are determined starting from the crank’s position at 12 o’clock, which occurs before the BDC of the beam pump. Therefore, it is essential to reorder the motion parameters to use the BDC as the starting point.

**Figure 4:**
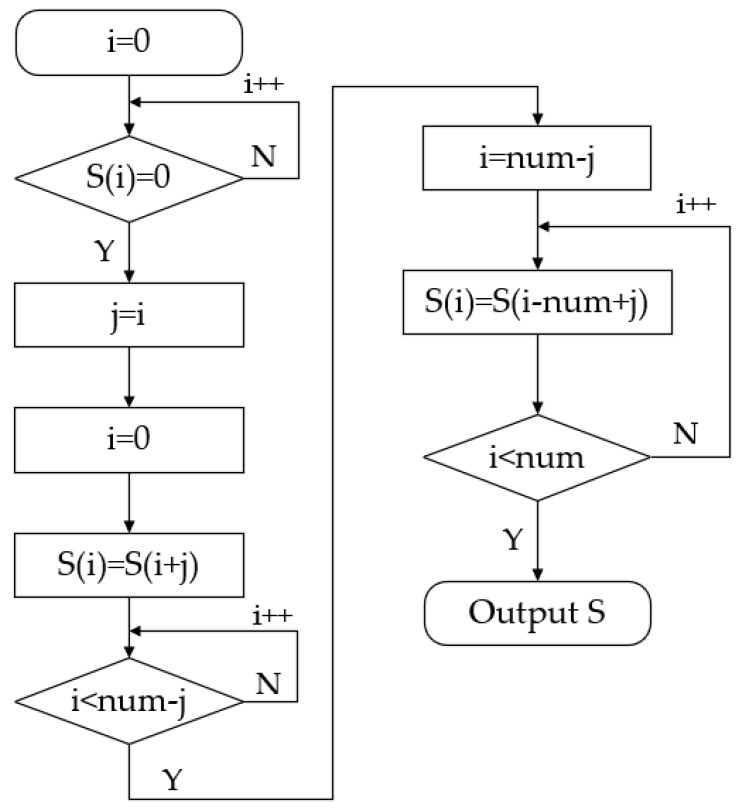
Flowchart of the motion parameter sorting algorithm.

**Figure 5:**
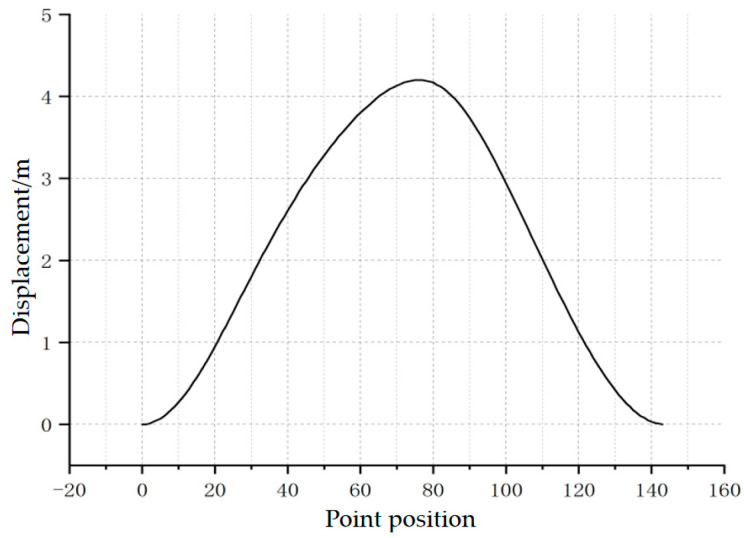
Polished rod displacement curve of the beam pump.

In Figure 4, I represents the sequence number of the motion parameters; S represents the displacement value at any given point; and num represents the total number of motion parameters, Figure 5 presents the displacement curve after correction.

## 3. Polished Rod Dynamometer Card Characteristic Point and Friction Load Identification Method

### 3.1. Method for Identifying Characteristic Points on the Polished Rod Dynamometer Card

**Figure 6:**
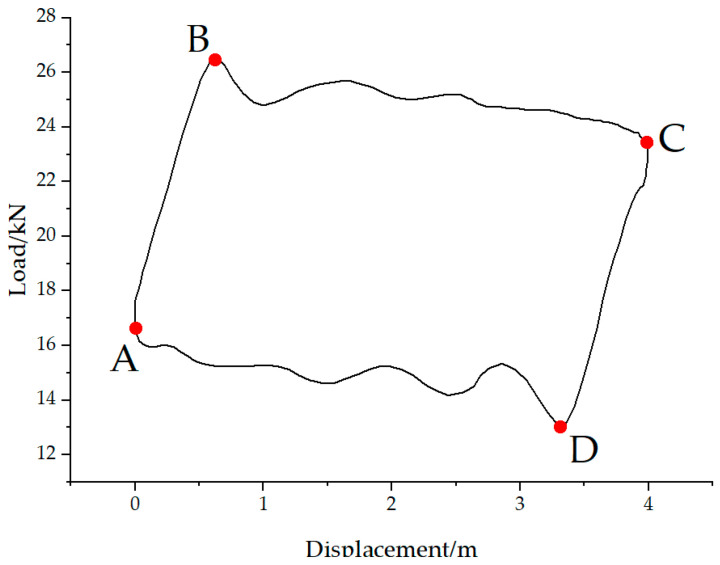
Four characteristic points on the polished rod dynamometer card.

The four characteristic points A, B, C, and D on the polished rod dynamometer card correspond to the BDC, the endpoint of the upward stroke rod deformation, the TDC, and the endpoint of the downward stroke rod deformation, as shown in Figure 6. Rapid identification of these four characteristic points on the polished rod dynamometer card serves the following purposes:

(1) The TDC and BDC of the beam pump are the points where the direction of the polished rod velocity changes. These points provide information about the friction load on the polished rod DC, helping to eliminate the impact of friction loads on the inversion of the polished rod DC to the downhole pump DC;
(2) The four characteristic points on the polished rod DC correspond to characteristic points on the pump DC. These points are crucial for solving key information related to production measurement and dynamic fluid level calculation in the pump dynamometer card.

In the identification of characteristic points on the polished rod DC, many researchers have utilized methods such as the five-point slope method or machine learning techniques. However, these methods often encounter problems with inaccurate identification of characteristic points, and their calculation processes can be quite complex. This complexity hinders their widespread adoption in engineering applications. This paper presents a new method that relies on the rate of change in the polished rod load and the corrected displacement curve, facilitating the rapid identification of four key characteristic points on the polished rod DC.

**Figure 7:**
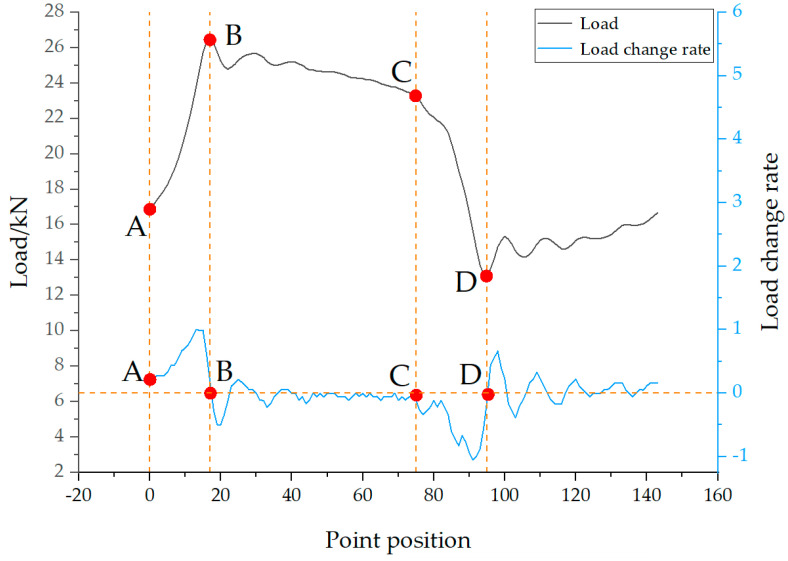
Positions of characteristic points in the load rate of change curve.

Qualitative Analysis of the Four Characteristic Point Locations:

According to the analysis of the polished rod DC in Figure 6, points A and C correspond to the minimum and maximum values of the polished rod displacement, respectively. Therefore, the time indices of points A and C can be rapidly determined by locating the minimum and maximum of the corrected displacement data. Points B and D represent the endpoints of elastic deformation in the upward and downward strokes of the rod string. Before these points, the load values change approximately linearly. B and D correspond to the peak and valley of the oscillatory load response of the rod string after vibration initiation. From the perspective of the rate of change in load, both points B and D are located where the load rate of change is close to zero. Specifically, the region before point B shows the maximum positive rate of change, while the region before point D exhibits the minimum (most negative) rate of change, as illustrated in Figure 7.

**Figure 8:**
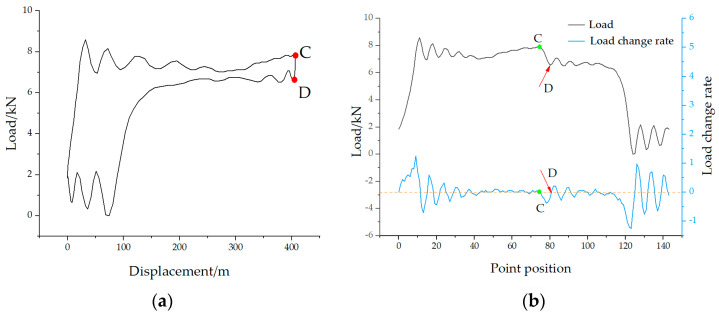
Incorrect characteristic point calibration in polished rod dynamometer card with insufficient fluid supply for (**a**) insufficient fluid supply dynamometer card and (**b**) incorrect characteristic point calibration.

In actual production, the well conditions of the beam pump are complex and variable, leading to different shapes of the polished rod DC. Especially in cases of insufficient fluid supply and gas influence, special situations may arise that cause errors in identifying point D, as shown in Figure 8, which represents a DC under insufficient fluid supply. Using the above method, in Figure 8a,b, multiple local valleys appear after point C, which interferes with the recognition and positioning of point D.

**Figure 9:**
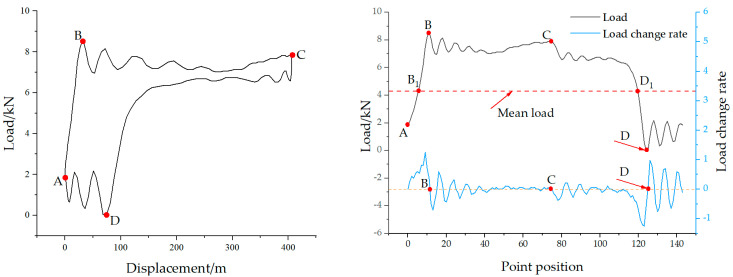
Correct characteristic point calibration in polished rod dynamometer card with insufficient fluid supply.

To address this issue, the average load method is employed to eliminate such interference in characteristic point identification. Specifically, the mean load is calculated as the average of the maximum and minimum polished rod loads within a complete stroke cycle. In addition, when the DC contains significant noise, a five-point moving average filter is applied to smooth the data. As mentioned earlier, the time indices of points A and C correspond to the minimum and maximum values of the polished rod displacement and can thus be quickly determined. To identify point B on the polished rod DC, the first step is to locate the intersection point (denoted as B1) between the mean load and the load curve, as shown in Figure 9. Based on a large number of DCs, it has been observed that the load value at point B typically increases, then decreases, and finally stabilizes. Accordingly, the rate of change in load transitions from positive to negative near this point. Therefore, the actual time index of point B corresponds to the first zero-crossing of the load rate of change after point B1. The identification of point D follows a similar approach. For a normal DC, the load at point D is typically lower than the mean load. First, the intersection between the mean load and the load curve is located and marked as point D1. The load behavior around point D generally decreases, then increases, and gradually stabilizes. Thus, the load rate of change near point D1 transitions from negative to positive. The time index at which this zero-crossing occurs corresponds to the location of characteristic point D on the displacement-load curve. By applying this method, characteristic points under various well conditions, including insufficient fluid supply, can be accurately identified, as illustrated in Figure 9.

### 3.2. Friction Load Identification Method

Researchers, both nationally and internationally, have conducted extensive investigations into the conversion of polished rod DCs into pump DCs [29]. The pump dynamometer card is vital for the digitalization of beam pump wells. In this conversion process, the Gibbs one-dimensional wave equation is typically employed [30]. However, this approach often neglects the influence of friction load, particularly when converting wax deposition DCs to pump DCs. Such oversight can result in an overestimation of production and fluid level data within the resulting pump DC. Therefore, it is crucial to implement strategies that minimize the effects of friction load before carrying out the conversion.

**Figure 10:**
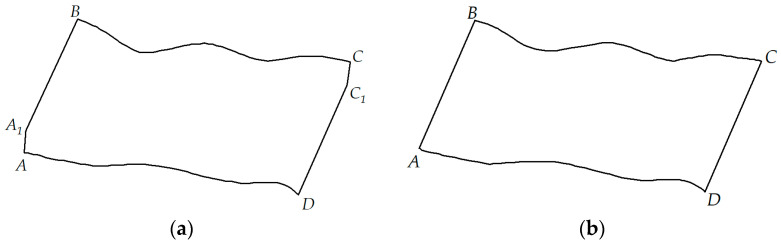
Polished rod dynamometer cards for (**a**) polished rod dynamometer card considering friction load and (**b**) ideal dynamometer card without friction load.

The polished rod DC provides information about the friction load. Points A and C represent the reversal points of the polished rod’s velocity. It is important to note that the friction load is inversely related to the direction of the velocity, as illustrated in Figure 10a.

Analyzing the polished rod dynamometer card in Figure 10a, near points A and C, the DC will show characteristics at points $A_{1}$ and $C_{1}$. These two points reflect the proportion of friction load in the polished rod DC. Assuming that the friction load is equal during the upstroke and downstroke of the beam pump, eliminating the impact of the load information at points $A_{1}$ and $C_{1}$ will yield the ideal polished rod DC shown in Figure 10b. The calculation method for the friction load is:

(4) $$P_{f}=\left(\frac{P_{A1}-P_{A}}{2}+\frac{P_{C}-P_{C1}}{2}\right)/2$$

Theoretical calculations show that by processing the polished rod DC using the above method, the influence of friction load on the conversion to the pump DC is greatly suppressed, resulting in higher theoretical calculation accuracy.

## 4. Polished Rod Dynamometer Card to Pump Dynamometer Card Conversion Model

**Figure 11:**
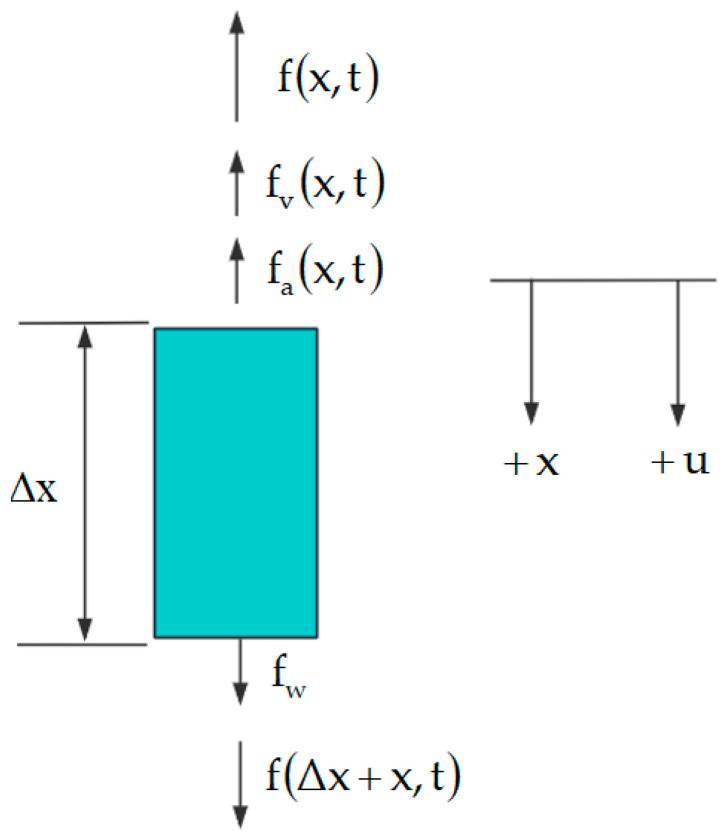
Force on the micro-element of the rod column.

As illustrated in Figure 11, let us examine micro-element Δx of the rod string, analyzing it from both the temporal and spatial perspectives. The spatial direction is defined as positive downward. The forces acting upon the micro-element include its weight, inertial force, damping force, and internal force.

In Figure 11, $f_{w}$ is the weight of the micro-element of the rod string; $f_{a}\left(x,t\right)$ is the inertial force of the micro-element of the rod string; $f_{v}\left(x,t\right)$ is the damping force of the micro-element of the rod string; and $f\left(x,t\right)$ and $f\left(\Delta x+x,t\right)$ are the internal forces within the rod string micro-element. The expressions for these forces are as follows:

(5) $$\left\{\begin{array}{l} f_{w}=\rho_{r}Ag\Delta x\\ f_{a}(x,t)=\rho_{r}A\Delta x\frac{\partial^{2}u(x,t)}{\partial t^{2}}\\ f_{v}\left(x,t\right)=v\Delta x\frac{\partial u\left(x,t\right)}{\partial x}\\ f(x,t)=EA{\left.\frac{\partial u(x,t)}{\partial x}\right|}_{x}\\ f(x+\Delta x)t)=EA{\left.\frac{\partial u(x,t)}{\partial x}\right|}_{x+\Delta x} \end{array}\right.$$

From the figure, based on the force equilibrium relationship, we have:

(6) $$f(x+\Delta x,t)-f(x,t)=f_{a}(x,t)+f_{\nu }(x,t)-f_{w}$$

After organizing the forces, we obtain the one-dimensional wave equation:

(7) $$\frac{\partial^{2}u(x,t)}{\partial t^{2}}=c^{2}\frac{\partial^{2}(x,t)}{\partial x^{2}}-\nu \frac{\partial (x,t)}{\partial t}+g$$

In the equation, μ is the viscosity of the well fluid, Pa⋅s; g is the gravitational acceleration, ${m/s}^{2}$; ν is the viscosity coefficient, $\nu =\frac{\mu }{\rho_{r}A_{r}}$; c is the wave speed, $c=\sqrt{\frac{E}{\rho }}$; $\rho_{r}$ is the density of the steel rod, 7850 kg/m^3^; d is the diameter of the polished rod, mm; $A_{r}$ is the cross-sectional area of the polished rod, mm^2^; E is the elastic modulus of the polished rod, $2.01\times 10^{11}\mathrm{Pa}$; and L is the pump depth, in meters.

The wave equation contains the second derivative of displacement with respect to time and the second partial derivative of displacement with respect to position. To solve the wave equation, two initial conditions and two boundary conditions are required.

For the same level of the rod string, divide the rod string into m units along the axis with equal step lengths, where the step length is Δx, and each unit is labeled with index *i*, where *i* = 0, 1, 2, 3, …, m. The time is divided into n steps with equal time intervals, where the time step is Δt, and each time point is labeled with index *j*, where *j* = 0, 1, 2, 3, …, n. The displacement at the i-th node on the rod string at time *j* is represented as $u_{i,j}$.

Using Newton’s forward interpolation formula, we obtain Equations (8) and (9):

(8) $${\left.\frac{\partial u}{\partial t}\right|}_{i,j}=\frac{u_{i,j+1}-u_{i,j}}{\Delta t}$$

(9) $${\left.\frac{\partial u}{\partial t}\right|}_{i,j}=\frac{u_{i,j}-u_{i,j-1}}{\Delta t}$$

From Equations (8) and (9), the central difference interpolation is obtained, as shown in Equation (10):

(10) $${\left.\frac{\partial^{2}u}{\partial t^{2}}\right|}_{i,j}=\frac{u_{i,j+1}-2u_{i,j}+u_{i,j-1}}{\Delta t^{2}}$$

Similarly, the second-order central difference formula for *u* with respect to *x* can be obtained, as shown in Equation (11):

(11) $${\left.\frac{\partial^{2}u}{\partial x^{2}}\right|}_{i,j}=\frac{u_{i+1,j}-2u_{i,j}+u_{i-1,j}}{\Delta x^{2}}$$

By substituting Equations (8)–(11) into Equation (7), the displacement equation for the homogeneous segment of the rod string is obtained as:

(12) $$u_{i+1,j}=\left(\frac{u_{i,j+1}-2u_{i,j}+u_{i,j-1}}{\Delta t^{2}}+\nu \frac{u_{i,j+1}-u_{i,j}}{\Delta t}-g\right)\frac{\Delta x^{2}}{c^{2}}+2u_{i,j}-u_{i-1,j}$$

The first layer of the difference grid represents the polished rod displacement, and the second layer of displacement can be calculated according to Hooke’s law. The first layer of the load is denoted as *f*_0,*j*_, which represents the polished rod load, and is given by:

(13) $$f_{0,j}=EA{\left.\frac{\partial u}{\partial x}\right|}_{0,j}=EA\frac{u_{1,j}-u_{0,j}}{\Delta x}$$

By transforming Equation (13), the displacement of the second layer can be obtained as follows:

(14) $$u_{1,j}=\frac{f_{0,j}\cdot \Delta x}{EA}+u_{0,j}$$

Starting from the third layer, during the displacement calculation process using Equation (11), it is necessary to use the difference grid form $u_{i,0}$ to $u_{i,n+1}$ compute the displacement at each layer’s time point. This requires applying the periodic boundary conditions, as follows:

(15) $$u_{i,0}=u_{i,n},\;u_{i,1}=u_{i,n+1}$$

**Figure 12:**
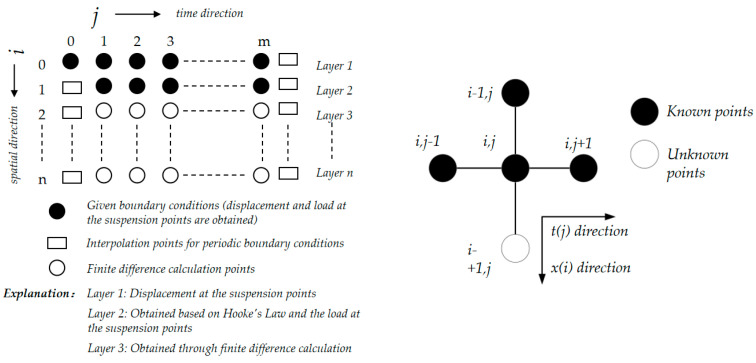
Grid diagram for solving the one-dimensional wave equation using the difference method.

Thus, by using the difference method, the displacements at different layers can be obtained. Then, by applying Hooke’s law, the loads at different time points of each layer can be determined. This allows for the generation of the dynamometer card at different positions of the rod string (with displacement as the horizontal axis and load as the vertical axis). The grid diagram for solving the wave equation is shown in Figure 12.

In the process of solving the pump DC using the difference method, the required well condition parameters are shown in Table 1.

Due to the complex and variable downhole conditions, the pump DC exhibits different shape characteristics depending on the polished rod DC. In this study, abnormal working conditions that affect the production measurement of the pump DC, such as self-flow, pump valve sticking, severe wear of the pump barrel inner wall, rod breakage, gas locking, and pump sticking, are first excluded. These conditions can be diagnosed and ruled out through the analysis of the polished rod DC.

## 5. Polished Rod Dynamometer Card Calibration and Production Calculation Model

### 5.1. Polished Rod Dynamometer Card Calibration Model

In Section 2 of this paper, we discussed the phenomena of zero drift and temperature drift associated with the polished rod dynamometer sensor. This section will now focus on the online calibration of the polished rod load, as well as the methods used for production calculations and liquid level measurement.

Assumptions:

(1) During the upward stroke, the traveling valve is closed, and the upper part is filled with well fluid. The gravity of the rod string in the liquid column and the oil mass on the plunger in the wellbore are known parameters. This value will be used as a reference for the calibration of the dynamometer card and the calculation of rod string liquid friction load.
(2) The polished rod dynamometer sensor does not lose elasticity.
(3) To simplify the load correction model, this study assumes that the average inertial load of the sucker rod over a full stroke cycle is approximately zero. Since the acceleration directions during the upstroke and downstroke are opposite, the inertial effects tend to cancel each other out.

The polished rod load of a beam pumping unit is essentially measured by a load sensor. When the load at the polished rod changes, slight deformation occurs in the sucker rod, and the load sensor converts this physical deformation into an electrical signal. The polished rod load can then be derived from this signal. As shown in Equation (2) of this paper, F represents the load value measured by the sensor. When the coefficients k, b experience drift, the measured load becomes only a relative value. Therefore, we denote k, b as $T_{f}$, $P_{0f}$, respectively, and the actual value of the polished rod load can be expressed by Equation (16). From Equation (2), it can be seen that the load measured by the load sensor is denoted as F measured. When the coefficient k, b experiences drift, the measured polished rod load is only a relative value. Therefore, the actual value of the polished rod load can be written as Equation (16):

(16) $$P_{m}=T_{f}\cdot F+P_{0f}$$

In Equation (16), $T_{f}$ is the temperature drift correction coefficient and $P_{0f}$ is the zero drift correction coefficient.

Under the assumed conditions, the average load in the polished rod DC during the upward stroke (from point B to point C) and the downward stroke (from point D to point A) is approximately equal to the load value in the static DC.

(17) $$\begin{array}{l} \bar{P_{mu}}=P_{s}+P_{l}+P_{o}F-P_{t}F\\ \;=\rho_{s}gf_{s}L+\rho_{l}g\left(F-f_{s}\right)L-\rho_{l}gh_{sd}F+P_{o}F-P_{t}F\\ \;=f_{s}L\left(\rho_{s}-\rho_{l}\right)g+F\left(L-h_{sd}\right)\rho_{l}g+P_{o}F-P_{t}F\\ \;=P_{s}^{\prime }+P_{l}^{\prime }+P_{o}F-P_{t}F \end{array}$$

In Equation (17), $\rho_{s}$ is the density of the sucker rod material, kg/m^3^; $\rho_{l}$ is the density of the produced fluid, kg/m^3^; $f_{s}$ is the cross-sectional area of the sucker rod, m^2^; F is the cross-sectional area of the pump plunger, m^2^; L is the length of the sucker rod or depth to the pump, m; $h_{sd}$ is the pump submersion depth, m; $P_{l}^{\prime }$ is the weight of the oil column above the dynamic liquid level in the well, with a cross-sectional area equal to the plunger area, N; $P_{s}^{\prime }$ is the weight of the sucker rod string in the liquid column, N; $P_{o}$ is the wellhead oil pressure, Pa; and $P_{t}$ is the casing pressure, Pa.

(18) $$\bar{P_{md}}=P_{s}^{\prime }$$

Combining Equations (16)–(18), we obtain:

(19) $$\left\{\begin{array}{l} \bar{P_{mu}}T_{f}+P_{0f}=\bar{P_{tu}}\\ \bar{P_{md}}T_{f}+P_{0f}=\bar{P_{td}} \end{array}\right.$$

In Equation (19), $\bar{P_{mu}}$ is the measured average load during the upstroke, kN; $\bar{P_{md}}$ is the measured average load during the downstroke, kN.

Since the values of $\bar{P_{mu}}$ and $\bar{P_{md}}$ can be obtained from the load sensor, and the values of $\bar{P_{tu}}$ and $\bar{P_{td}}$ can be calculated from the well condition parameters of the beam-pumping well, the temperature drift correction coefficient ($T_{f}$) and zero drift correction coefficient ($P_{0f}$) can be determined using Equation (19). Once obtained, these coefficients are used to correct the entire measured polished rod load profile.

### 5.2. Production and Fluid Level Calculation Model

In Equation (19), the suspension point load correction model assumes that the well’s dynamic liquid level or settling depth is a known parameter. However, in the practical application of digitizing oil well power curves, determining the dynamic liquid level is a crucial task. By integrating the methodologies for calculating pump curves and performing online corrections of suspension point power curves discussed in Section 4, the dynamic liquid level can be derived using an iterative difference method for the pump curve. This approach ensures that the predicted dynamic liquid level aligns closely with the calculated dynamic liquid level, remaining within an acceptable margin of error. Consequently, this facilitates the determination of the dynamic liquid level in the oil well, as well as the correction of the load sensor’s temperature drift and zero drift coefficients. The calculation process is outlined below.

**Figure 13:**
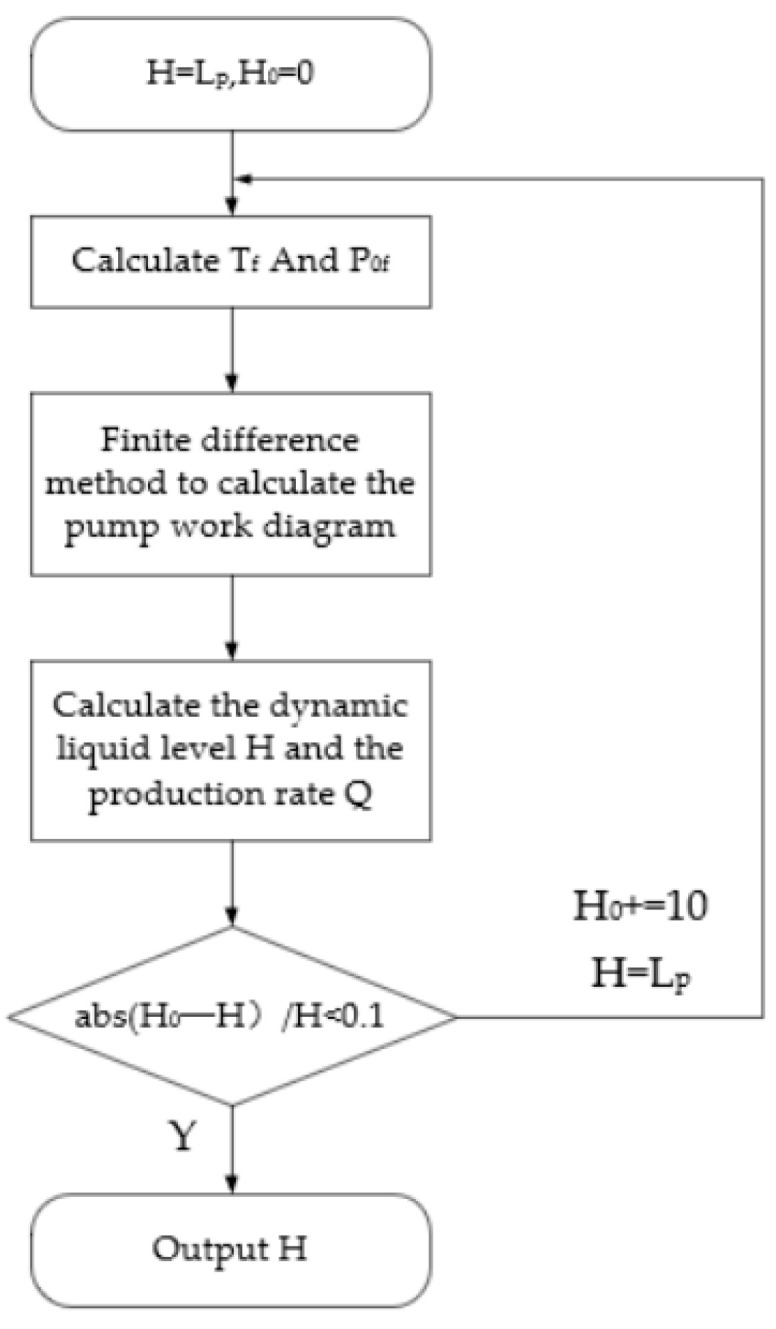
Flowchart of the iterative method for solving the pump power curve, dynamic liquid level, and production rate.

In Figure 13, the process involves predicting and calculating the dynamic liquid level. The fundamental mathematical model used to iterate the well’s dynamic liquid level is presented in Equations (20) and (21). By utilizing the corrected suspension point DC to convert the pump DC, we can determine the load difference during both the upstroke and downstroke of the pump curve. Using these load characteristics, we can then calculate the depth of the dynamic liquid level in the oil well.

(20) $$\Delta P=\bar{P_{pu}}-\bar{P_{pd}}$$

(21) $$H=\frac{\Delta P-\bar{P_{o}}F+\bar{P_{t}}F}{\rho_{l}gF}$$

In Equation (21), $\bar{P_{pu}}$ is the average load on the upstroke in the pump power curve, kN; and $\bar{P_{pd}}$ is the average load on the downstroke in the pump power curve, kN.

The oil well production rate is related to the sucker rod stroke, plunger diameter, and other factors. However, in actual production, the pumping unit inevitably experiences leakage, and the production rate is calculated as follows:

(22) $$Q_{t}=1440\cdot \left(S_{pe}\cdot F-\Delta Q\right)\cdot N$$

In Equation (22), $S_{pe}$ is the effective stroke of the pump plunger, m; F is the plunger cross-sectional area, m^2^; ΔQ is the leakage volume per stroke of the pump, m^3^ per stroke; and N is the strokes per minute, min^−1^.

The leakage in the oil well mainly considers the leakage between the plunger and the pump barrel. The leakage per stroke ΔQ is:

(23) $$\Delta Q=\frac{\pi D_{p}\delta^{3}\left(P_{d}-P_{c}\right)}{12\mu l_{Z}}\left[1+\frac{3}{2}{\left(\frac{e}{\delta }\right)}^{2}\right]-\frac{\pi D_{p}\delta \bar{v_{u}}}{2}$$

(24) $$P_{d}=\rho_{l}gh_{sd}+P_{t}$$

(25) $$P_{c}=\rho_{l}gh_{pd}+P_{o}$$

In Equation (25), the terms are defined as follows: $D_{p}$ is the plunger diameter of the pumping unit, m; δ is the average concentric gap between the plunger and pump barrel, m; $P_{d}$ is the pump discharge pressure, kPa; $P_{d}$ is the pump intake pressure, kPa; μ is fluid dynamic viscosity, kPa·s; $l_{Z}$ is the plunger length, m; e is the eccentricity, m; $\bar{v_{u}}$ is the average upward stroke velocity, m/s; and $h_{pd}$ is the pump depth, m.

## 6. Results and Discussion

Five typical well indicator diagrams were selected from beam-pumping wells in the Liaohe Oilfield to validate the effectiveness of the proposed method under actual field conditions. The well parameters are summarized in Table 2.

**Figure 14:**
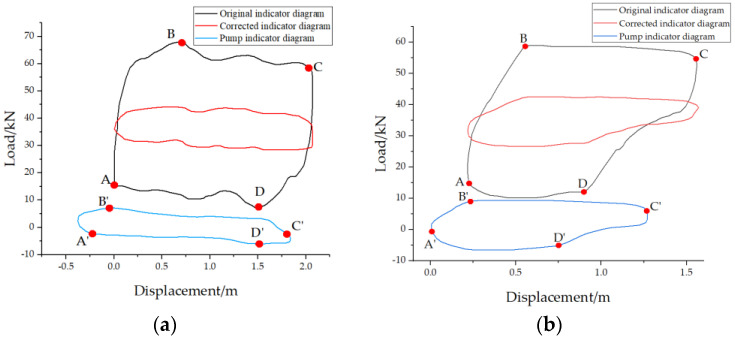

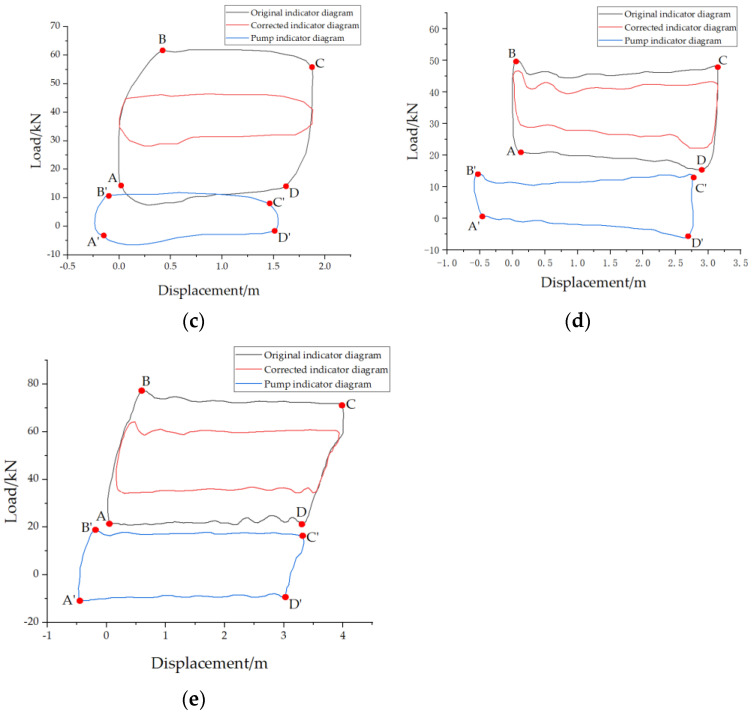
Suspension point power curve and pump power curve before and after correction for (**a**) well 1, (**b**) well 2, (**c**) well 3, (**d**) well 4, and (**e**) well 5.

The comparison results of the online calibrated suspension point power curve and pump power curve are shown in Figure 14a–e.

The comparison results of the measured data and predicted data for the five wells are shown in Table 3.

As shown in Table 3, when using the obtained pump performance curve to calculate production and dynamic fluid level, the prediction accuracy can exceed 90%. To further evaluate the predictive performance of the proposed method, a large number of wells were analyzed. Due to space limitations, five representative wells were selected for comparison between the measured and predicted values, followed by a statistical error analysis of dynamic fluid level and daily production.

The formula for the mean absolute error (MAE) is:

(26) $$MAE=\frac{1}{n}\sum_{i=1}^{n}\left|x_{i}-{\hat{x}}_{i}\right|$$

In Equation (26), $x_{i}$ represents the *i*-th measured (actual) value, and ${\hat{x}}_{i}$ represents the *i*-th predicted value.

The formula for the mean absolute percentage error (MAPE) is:

(27) $$MAPE=\frac{1}{n}\sum_{i=1}^{n}\left|\frac{y_{i}-{\hat{y}}_{i}}{y_{i}}\right|\times 100\%$$

In Equation (27), $y_{i}$ represents the *i*-th measured (actual) value, and ${\hat{y}}_{i}$ represents the *i*-th predicted value.

By substituting the measured and predicted values of fluid level and production from Table 3 into the formulas, the following results were obtained: for fluid level prediction, the MAE is 70.03 m, and the MAPE is 7.17%; for production prediction, the MAE is 0.67 m^3^/d, and the MAPE is 6.40%. These results indicate that the proposed method achieves high prediction accuracy for both fluid level and production, fully meeting the error tolerance requirements for engineering applications. In addition, the method demonstrates good adaptability under different well depths and production rates, further validating the robustness of the algorithm.

Although the predicted values of production and dynamic fluid level show a high level of agreement with field measurements, certain discrepancies still exist. These differences can be attributed to multiple factors. For production errors, the primary reason lies in the varying degrees of wear in the pump barrel after long-term operation. This leads to differences in the concentric clearance between the plunger and barrel across different wells, resulting in non-uniform leakage volumes per stroke. Since the model currently uses a theoretical or average leakage value, this variation can cause discrepancies between the predicted and actual production rates. For dynamic fluid level errors, the current measurement method is acoustic liquid level detection, which inherently contains certain uncertainties. These arise from factors such as echo signal interference, attenuation in gas phases, and subjective bias in manual interpretation. These issues directly affect the accuracy of the measured dynamic fluid level, thereby contributing to deviations from the model-predicted values.

To further improve the accuracy of the model predictions, the following strategies will be considered in future work:

(1) Developing a dynamic leakage coefficient model that adjusts the estimated plunger-barrel leakage in real time based on well history data, pump specifications, and operating conditions.
(2) Applying parameter self-adaptive optimization algorithms, such as model correction methods based on machine learning, to iteratively adjust key parameters, including friction correction coefficients and leakage coefficients, based on feedback from actual production and fluid level data.

In practical implementation, the dynamic leakage coefficient model can be constructed by integrating historical dynamometer cards, stroke frequency, tubing pressure, and temperature data to establish a multi-parameter correlation with per-stroke leakage. This will enable real-time updates of leakage estimates as operating conditions change. Additionally, the use of supervised machine learning algorithms—such as gradient boosting decision trees (GBDT) or artificial neural networks (ANNs)—can help establish an adaptive correction model. By training on historical data where measured production and fluid level are known, the model can learn the nonlinear relationship between predicted errors and influencing parameters (e.g., friction, fluid properties, depth). These techniques allow automatic adjustment of coefficients in the prediction model, effectively reducing the cumulative error caused by static assumptions and improving prediction robustness across diverse well conditions.

## 7. Conclusions

Based on the theoretical research findings presented in this paper and a comparative analysis with practical engineering applications, the effectiveness of the algorithms for the rapid identification of feature points in the sucker point DC, friction load information, pump DC calculations, and fluid level and production predictions has been validated. These results hold significant theoretical and engineering value for the digitization of oilfields. The following conclusions can be drawn:

(1) A method for quickly identifying the four characteristic points in the sucker point load diagram based on the load variation rate has been proposed, which provides a foundation for subsequent load diagram correction and conversion of sucker point load diagrams to pump load diagrams.
(2) By combining the sucker point DC with wellbore parameters and solving the one-dimensional wave equation and load characteristics using the finite difference method, an online calibration theory model for load sensor zero drift and temperature drift has been proposed. Additionally, a method for extracting friction load information from sucker point velocity reversal points has been developed, providing accurate theoretical calculation methods and technologies for oilfield digitization applications.
(3) An iterative finite difference method for solving the one-dimensional wave equation has been used to obtain the online calibration of temperature drift and zero drift coefficients. This has enabled the calculation of oil well production and fluid levels based on pump DC. The results show that the accuracy of the oil well production and fluid level prediction exceeds 90% when compared to actual well production parameters.

## Figures and Tables

**Table 1:** Required well condition parameters.

Parameter	Unit	Parameter	Unit
Polished Rod Diameter	mm	Plunger Diameter	mm
Polished Rod Length	m	Pump Depth	m
Water Cut	%	Oil Pressure	MPa
Casing Pressure	MPa	Stroke Rate	min^−1^

**Table 2:** Well parameters.

Parameter	Well 1	Well 2	Well 3	Well 4	Well 5
First-Stage Rod Length (m)	518.81	1106.19	1200.20	1456.86	9.14
First-Stage Rod Length (mm)	22	22	22	19	28
Second-Stage Rod Length (m)	697.27	—	—	—	810.02
Second-Stage Rod Diameter (mm)	19	—	—	—	28
Third-Stage Rod Length (m)	200.46	—	—	—	—
Third-Stage Rod Diameter (mm)	22	—	—	—	—
Oil Pressure (Mpa)	0.61	0.67	0.42	0.38	0.44
Casing Pressure (Mpa)	0.62	0.70	0.70	0.40	0.63
Pump Diameter (mm)	28	44	44	38	83
Pump Depth (m)	1434.97	1106.19	1200.20	1471.92	830.71
Water Cut (%)	20.3	64.2	28.5	72.0	99.8
Stroke (m)	2.07	1.58	1.88	3.16	4.01
Stroke Frequency (min^−1^)	5.64	1.87	2.93	3.48	2.82

**Table 3:** Statistical table of measured and predicted values for production and fluid level.

Well ID	Measured Fluid Level (m)	Predicted Fluid Level (m)	Accuracy (%)	Measured Production (m^3^/d)	Predicted Production (m^3^/d)	Accuracy (%)
1	1011.08	1099.72	91.23	6.69	7.19	92.53
2	866.83	922.16	93.62	3.16	2.91	92.09
3	1087.63	998.93	91.84	6.94	7.31	94.67
4	1168.89	1256.88	92.47	17.57	16.32	92.89
5	590.14	560.63	94.99	23.59	24.57	95.85

## Data Availability

Data are contained within the article.
